# Epidemiology of HIV-associated tuberculosis (TB) co-infection in Krasnoyarsk region, Russian Federation

**DOI:** 10.1186/1758-2652-13-S4-P193

**Published:** 2010-11-08

**Authors:** TA Chaychuk, NU Gankina, OG Nikonova

**Affiliations:** 1 Regional Center of AIDS Prevention, consultative and treatment of patients with HIV, Krasnoyarsk, Russian Federation

## Background

Incidence of HIV-associated tuberculosis (TB) co-infection is increasing in Krasnoyarsk region as well as in RF. Total number of registered TB/HIV new cases has increased from 7 in 2000 to 170 in 2009. Mortality rate of patients with HIV/TB co-infection has increased: 2004-7 cases, 2008-40, 2009-48. On January 1st, 2010 we have 407 TB/HIV co-infected patients, which makes 6% from the HIV+ patients under supervision (n=6801) in our region.

## Methods

A retrospective chart review was conducted on HIV-positive patients diagnosed with TB in 2009. All patients were HIV-positive and had confirmed diagnosis of mycobacterial tuberculosis, or clinically presumptive diagnosis of TB with response to anti-tuberculosis therapy.

## Summary of results

A total of 170 HIV/TB co-infection patients were identified. The general characteristics of the patients are: male (75%), median age 28 years old [1;55] when HIV has been diagnosed and 33 years old [5;56] at the time of TB diagnosis. HIV was acquired vertically - 0,6%, sexually in 22% of the cases, and through intravenous drug injections - 74%. At the time of TB diagnosis, 70 (40%) had a CD4 count of less than 200. 23% had a baseline HIV viral load greater than 100,000 copies/ml, median VL were 338787 copies/ml. 9,4% of cases had a history of TB prior to HIV diagnosis. 14% had TB as the primary AIDS-defining event; 82 patients (48%) had pulmonary disease, 47 patients (28%) presented with extra-pulmonary disease and of these, 22 (13%) had disseminated disease. 28% cases had extra-pulmonary TB involvement and are related to lower CD4 count (median 189/uL vs. 388/µL, p<0.001), and mortality rate. 28 patients (16,5%) had multi-drug resistant TB. 16,5% patients received antiretroviral treatment (ARV) at the time of TB diagnosis. Anti-TB treatment was associated with ARV on 39% (6% PI and 33% NNRTI). Out of 170 HIV/TB patients 35 (21%) were died. Risk of death is related to severity of immunosuppression and TB diagnostic time (median CD4 count 353 in survivors vs 79 in dead, p<0.001). The chronic hepatitis C was found in 69% of patients with HIV/TB infections.

## Conclusions

1. HIV/TB co-infection has a high incidence rate due to delayed verification of TB infection, as a result the prognosis is poor; 2. Risk factors for such patients are low CD4 cell count and extra-pulmonary involvement; 3. The high prevalence of the chronic hepatitis C causes the hepatotoxicity increasing during ART and antitubercular therapy for patients with HIV/TB infections.

